# Astragaloside IV regulates FOXM1 deubiquitination to ameliorate trophoblast damage caused by high glucose

**DOI:** 10.1186/s41065-025-00465-w

**Published:** 2025-06-13

**Authors:** Fan Li, Xiaofang Zhao

**Affiliations:** Department of Obstetrics and Gynecology, Ankang People’s Hospital, No.38 Jiangbei Avenve, Hanbin District, Ankang, 725000 China

**Keywords:** Astragaloside IV, Forkhead box protein M1, Hyperglycemic induction, Deubiquitination, Gestational diabetes mellitus

## Abstract

**Background:**

Gestational diabetes mellitus (GDM) is a common metabolic complication during pregnancy that poses significant risks to both the pregnant woman and her fetus. Astragaloside IV (Ast IV) belongs to the class of triterpenoid saponins and exhibits important physiological roles in various aspects, including antidiabetic, antioxidant, and antiviral effects. The main objective of this study is to investigate the effects of Ast IV on trophoblast damage caused by high glucose (HG) and its underlying mechanism of action.

**Methods:**

Cell viability was determined by the CCK8 assay. The levels of oxidative stress in cells were determined by lactate dehydrogenase (LDH), malondialdehyde (MDA), and reactive oxygen species (ROS) kits. Ferroptosis in cells was assessed by the iron content kit. Gene expression levels were detected by real-time quantitative reverse transcription PCR (qRT-PCR) and western blot. The protein stability of Forkhead box protein M1 (FOXM1) was determined by the cycloheximide (CHX) assay. The ubiquitination level of FOXM1 was detected by the immunoprecipitation assay.

**Results:**

Ast IV alleviated the inhibitory effect of HG on the proliferation of HTR-8/SVneo cells and reduced HG-induced oxidative stress and ferroptosis. Ast IV was able to decrease the ubiquitination of FOXM1, thereby ensuring the stability of its expression. The overexpression of FOXM1 significantly mitigated the inhibitory effect of HG on the viability of HTR-8/SVneo cells and concurrently decreased the occurrence of HG-induced oxidative stress and ferroptosis processes. Conversely, knockdown of FOXM1 diminished the protective effect of Ast IV on HTR-8/SVneo cells.

**Conclusions:**

Ast IV ameliorates HG-induced trophoblast injury by modulating deubiquitination of FOXM1, which provides a new insight into the treatment of GDM.

**Supplementary Information:**

The online version contains supplementary material available at 10.1186/s41065-025-00465-w.

## Introduction

Diabetes mellitus (DM) consists of three main types: type 1 diabetes (T1D), type 2diabetes (T2D), and a form that occurs during pregnancy, known as gestational diabetes mellitus (GDM) [1]. GDM represents a common metabolic issue arising during pregnancy and poses a considerable threat to adverse pregnancy outcomes for both the mother and her baby, both before and after birth [2, 3]. GDM was first elaborately described by the German scholar Bennewitz in 1824, and this information remains relevant today [4]. Following this, a series of in-depth case studies conducted by researchers in the United Kingdom and the United States have collectively highlighted the elevated perinatal mortality risk faced by women with GDM [4]. GDM also results in a variety of more serious health issues, including chronic hypertension, hypoglycemia, hypokalemia, obesity, and an increased risk of fetal disease and death, posing harm to both the mother and her fetus [5–7]. This stems from the fact that the amount of insulin produced by maternal β-cells is insufficient to adequately address the progressive rise in insulin resistance during pregnancy [8], causing a decrease in glucose uptake, an augmentation of gluconeogenesis in the liver, and an elevation in maternal glucose levels [9]. Currently, research on the pathogenesis of GDM and its potential therapeutic targets remains scarce.

Astragaloside IV (Ast IV), a saponin compound belonging to the triterpenoid class, extracted from the roots of the plant species Astragalus membranaceus, it is well-documented in numerous academic literature [10]. Studies show that Ast IV can significantly reduce the damage caused by numerous pathological states, such as Alzheimer’s disease (AD), Parkinson’s disease (PD), and cerebral ischemia [11, 12]. Ast IV demonstrates a wide range of bioactivities, including antidiabetic, antioxidant, antiviral, antimicrobial, anti-inflammatory, asthma-relieving, antifibrotic, immune-modulating, and cardioprotective functions [13, 14]. In animal models of subarachnoid hemorrhage, treatment with Ast IV significantly reduced cerebral oedema compared to the carrier control [15]. Additionally, Ast IV improved neurobehavioral outcomes and alleviated brain injury through its antioxidant and anti-apoptotic effects [16]. In diabetes research, Ast IV exhibited significant antidiabetic effects, optimizing metabolic profiles and attenuated abnormalities in adipose function in fructose-fed mice, thereby validating its efficacy in promoting metabolism [16]. Studies indicated that a high glucose (HG) environment accelerated cell death, resulting in a substantial elevation in the levels of reactive oxygen species (ROS) and glutathione in its oxidized form (GSSG), as well as enhanced lipid peroxidation in mitochondrial membranes [17]. In contrast, Ast IV reduced the concentration of reduced glutathione (GSH), decreased the volume of mitochondria, and simplified the structure of their internal ridges [17]. However, our understanding of the specific mechanism by which Ast IV reverses the effects of HG on cells is not yet sufficient.

Forkhead box protein M1 (FOXM1) is a transcription factor that has a pivotal role in pancreatic islet cells, where it is responsible for controlling the expression of genes intimately linked to insulin (INS) secretion and may potentially function as a target for regulation to enhance insulin synthesis and secretion [18]. Studies showed that diabetic patients with nephropathy, retinopathy, cardiovascular disease, diabetic foot ulcers (DFUs), and tumors exhibited abnormal expression patterns of FOXM1 [19, 20]. FOXM1 exerts its influence on DM and its complications by modulating the functional activities of a range of immune cells, including T cells, B cells, monocytes, macrophages, and dendritic cells [20, 21]. The regulation of FOXM1 expression and activity is governed by diverse signaling cascades and molecules, including the YAP1/Akt/Glycogen synthase kinase-3β (GSK3β) axis and the FOXM1-associated GSK3β pathway [22]. Thus, FOXM1 emerges as a pivotal regulator in the modulation of these crucial signaling cascades and molecular mechanisms underlying the development of diabetes and its associated complications.

Ubiquitination, as a crucial post-translational modification (PTM), holds immense importance within eukaryotic cells [23]. Multiple studies confirm that ubiquitination modification plays a crucial role in regulating FOXM1 protein during various disease processes [24, 25]. Research revealed that overexpression of RNF112 could significantly elevate the ubiquitination level of FOXM1, resulting in FOXM1 degradation and thereby inhibiting the proliferation, invasion, and metastasis abilities of gastric cancer cells [26]. Previous research demonstrated that aberrant FOXM1 expression drove the proliferation, invasion, and metastasis of breast cancer cells, with modulation of its ubiquitination levels serving as a potential regulatory mechanism influencing these malignant behaviors and, consequently, impacting disease progression and patient prognosis [27, 28]. However, currently, there is still limited knowledge about the specific role of FOXM1 regulated by ubiquitination in GDM.

GDM presents a significant threat to the health of both women and fetuses; however, effective treatments for GDM are still unavailable. Thus, conducting thorough research into potential therapeutic targets for GDM is extremely urgent and essential.

## Methods

### Cell culture

The human chorionic trophoblast cells (HTR-8/SV) were acquired from Keycell (Wuhan, China). Additionally, Ast IV and glucose were obtained from MedChemExpress (Shanghai, China). The HTR-8/SVneo cells were cultured in RPMI 1640 medium (Invitrogen, Waltham, MA, USA) supplemented with 10% fetal bovine serum (FBS) (Invitrogen) and a 1% penicillin-streptomycin mixture (Invitrogen), within a 37℃, 5% CO₂ incubator. Once the cells had reached a stable state, they were categorized into five distinct groups: the control group (with 5.5 mM glucose), the HG group (with 25 mM glucose), the HG + Ast IV 10 µM group (with 25 mM glucose + 10 µM Ast IV), the HG + Ast IV 20 µM group (with 25 mM glucose + 20 µM Ast IV), and the HG + Ast IV 40 µM group (with 25 mM glucose + 40 µM Ast IV). Prior to the addition of 25 mM glucose, the HTR-8/SV cells had been treated with Ast IV for 40 min beforehand [29].

### Viability assessment of cells

The viability assay of HTR-8/SV cells was performed using the cell counting kit-8 kit (CCK8, MedChemExpress). First, HTR-8/SV cells in the logarithmic growth phase were digested and then prepared into a single-cell suspension, with the cell concentration adjusted to 5 × 10³ cells/mL. The cell suspension was dispensed into 96-well plates at a volume of 100 µL per well. The 96-well plate was placed in an incubator set at 37℃ with 5% CO_2_ and incubated for 24 h to allow the cells to adhere fully to the plate. Subsequently, the original medium was carefully aspirated, and a new medium containing 10% CCK8 reagent was added to each well. Afterward, the 96-well plate was returned to the incubator and incubated for an additional 3 h under light-shielded conditions. After being incubated, the absorbance at 450 nm was measured.

### Measurement of lactate dehydrogenase (LDH) levels and malondialdehyde (MDA) content

The Lactate LDH Activity Assay Kit (Elabscience, Wuhan, China) and the MDA Colorimetric Assay Kit (cell samples) (Elabscience) were used to determine the levels of LDH and MDA in HTR-8/SV cells. Approximately 1 × 10^6^ HTR-8/SV cells were taken and were sonicated in phosphate buffered saline (PBS). After being centrifuged, the supernatant was extracted. The levels of LDH and MDA were measured following the guidelines outlined in the respective kits, respectively.

### Measurement of ROS levels

The ROS levels in HTR-8/SV cells were determined using the Reactive Oxygen Species Fluorometric Assay Kit (Elabscience). After the cell culture medium was removed, the well-conditioned HTR-8/SV cells were rinsed using medium devoid of serum, subsequently undergoing the assay as per the kit’s instructions.

### Determination of total iron content

The iron levels in HTR-8/SV cells were determined using the Cell Total Iron Colorimetric Assay Kit (Elabscience). Approximately 1 × 10^6^ HTR-8/SV cells were taken and were added to 0.2 mL of the special buffer provided in the kit. They were mixed well, lysed on ice, and spun at a speed of 4,000 revolutions per min for a period of 10 min in order to harvest the supernatant. Subsequently, the iron content in the supernatant was tested strictly according to the instructions provided in the kit.

### Western blot

The western blot assay confirmed the protein expression levels of glutathione peroxidase 4 (GXP4), solute carrier family 7 member 11 (SLC7A11), ferritin heavy polypeptide 1 (FTH1), arachidonate 12-lipoxygenase (ALOX12), and FOXM1 within all groups of HTR-8/SV cells. HTR-8/SV cells harvested from different treatments were used to extract cellular whole proteins with RIPA lysate (Proteintech, Wuhan, China) containing phosphatase (Proteintech) and protease inhibitors (Proteintech). The total protein content was determined using a bicinchoninic acid protein assay kit (Proteintech). Electrophoresis using a 10% sodium dodecyl sulfate-polyacrylamide gel (SDS-PAGE) was employed to separate the total protein, which was then transferred onto a polyvinylidene fluoride (PVDF) membrane (Millipore, Billerica, MA, USA) with a pore size of 0.45µM using the wet transfer method. The membrane with transferred proteins was incubated at room temperature with phosphate buffer + Tween + 5% bovine serum albumin for 2 h. The closed PVDF membranes were then incubated with anti-GXP4 antibody (ab116703, 1/5000, Abcam, Cambridge, UK), anti-SLC7A11 antibody (ab216876, 1/1500, Abcam), anti-FTH1 antibody (ab247418, 1/2000, Abcam), anti-ALOX12 antibody (ab168384, 1/3000, Abcam), anti-FOXM1 antibody (ab245309, 1/4000, Abcam), and anti-glyceraldehyde-3-phosphate dehydrogenase (GAPDH) antibody (ab9485, 1/2500, Abcam) for 1 h at 37℃. Following the incubation with the primary antibody, the membranes underwent a further incubation with the corresponding secondary antibody at a temperature of 37 for a duration of 1 h. Upon completion of this incubation period, the membranes were visualized using the Enhanced Chemiluminescence Chemiluminescent Detection Kit (Proteintech).

### RNA extraction and real-time quantitative reverse transcription PCR

RNA from HTR-8/SV cells was extracted using the Cytoplasmic and Nuclear RNA Purification Kit (Norgen Biotek Corp, Ontario, Canada). The quality and concentration of the RNA were determined spectrophotometrically after extraction. The RNA was converted using the Transcriptor First Strand cDNA Synthesis Kit (Roche, Vilvoord, Brussel, Belgium). The cDNA obtained from reversal, along with the primers and the ChamQ Universal SYBR qPCR Master Mix (Vazyme, Nanjing, China) were combined together. Subsequently, the mixture was added to the qPCR-specific octuplex (Vazyme). The mixture was then placed into the 7300 Real-Time PCR System (Applied Biosystems, Foster City, USA). Gene expression was normalized using GAPDH, and the 2^−ΔΔCt^ method was utilized to calculate gene expression. The primer sequences employed in the experiments were shown below: FOXM1, F 5’-CCTTCTGGACCATTCACCCC-3’ and R 5’-GATTCGGTCGTTTCTGCTGC-3’; GAPDH, F 5’-AGAAGGCTGGGGGCTCATTTG-3’ and R 5’-AGGGGCCATCCACAGTCTTC-3’. The details of the PCR reaction procedure were referred to in Supplementary Table 1.

### Protein stability assay

The protein stability of FOXM1 was determined using the cycloheximide (CHX) method. Cells were treated with 100 µg/mL of CHX (Merck, Darmstadt, Germany), and cell samples were collected at time points of 1 h, 2 h, 4 h, 8 h, and 12 h post-CHX treatment. These samples were then subjected to a western blot assay to detect protein levels. Three replicates were set up for each time point.

### Cellular transfection assay

The overexpression vector (pcDNA-FOXM1) and the small interfering RNA (si-FOXM1) for FOXM1 were acquired from Tsingke Biotechnology Co., Ltd. (Beijing, China); non-targeting siRNA (si-NC) and empty plasmid (pcDNA) were used as negative controls. The pcDNA-FOXM1 or si-FOXM1 was mixed proportionally with Lipofectamine™ 2000 (Invitrogen). The mixture was then allowed to incubate at room temperature for a duration of 20 min. HTR-8/SV cells, at a concentration of 1 × 10^5^ cells per mL during their logarithmic growth phase, were seeded into 6-well plates pre-filled with serum-free medium. Subsequently, the mixture was introduced to the HTR-8/SV cells and allowed to incubate under conditions of 37℃ and 5% CO_2_ for a period of 5 hours.

### Immunoprecipitation assay

The 20 µL of Protein A/G magnetic beads (Invitrogen) were placed into a centrifuge tube and washed three times with 500 µL of 1× PBS buffer (containing 0.1% BSA) (Invitrogen) for 5 min each time, with gentle rotation at 4℃. Subsequently, 1 µg of anti-FOXM1 antibody (ab245309, 1:5000, Abcam) or anti-ubiquitin antibody (ab277768, 1:1000, Abcam) was added, and the mixture was incubated with rotation at 4℃ for 4 h to form an antibody-magnetic bead complex. This complex was then combined with 500 µg of cell lysate (containing 1× RIPA buffer and protease inhibitor cocktail) and incubated overnight with rotation at 4℃. The magnetic beads were then washed five times with 1 mL of 1× PBS buffer (containing 0.1% Tween-20) (Invitrogen) for 5 min each time, with gentle rotation at 4℃. After the final wash, the beads were separated using a magnetic rack, and the supernatant was discarded. Two times SDS sample buffer (Invitrogen) was added to the precipitate, and the mixture was heated at 95℃ for 10 min to elute the bound proteins. The eluted proteins were separated by SDS-PAGE gel electrophoresis, transferred onto a PVDF membrane (Millipore), and subsequently analyzed by immunoblotting using either anti-FOXM1 antibody (ab245309, 1:5000, Abcam) or anti-ubiquitin antibody (ab277768, 1:1000, Abcam). The immunoblotting results were visualized by chemiluminescence.

### Data analysis

The statistical analysis of the data was conducted using the GraphPad Prism 7.0 statistical software (GraphPad Software, Boston, MA, USA), and the results were presented in the format of “mean ± standard deviation”. For the comparison of data between two groups, a two-tailed independent samples *t*-test was employed; for the comparison of data among three or more groups, a one-way analysis of variance (ANOVA) was conducted, followed by the application of the Tukey multiple comparisons correction method to control the false positive rate. The significance of statistics was regarded as * *P* < 0.05, ** *P* < 0.01, and *** *P* < 0.001.

## Results

### Ast IV inhibits the damage caused by HG in HTR-8/SVneo cells

**Fig. 1:**
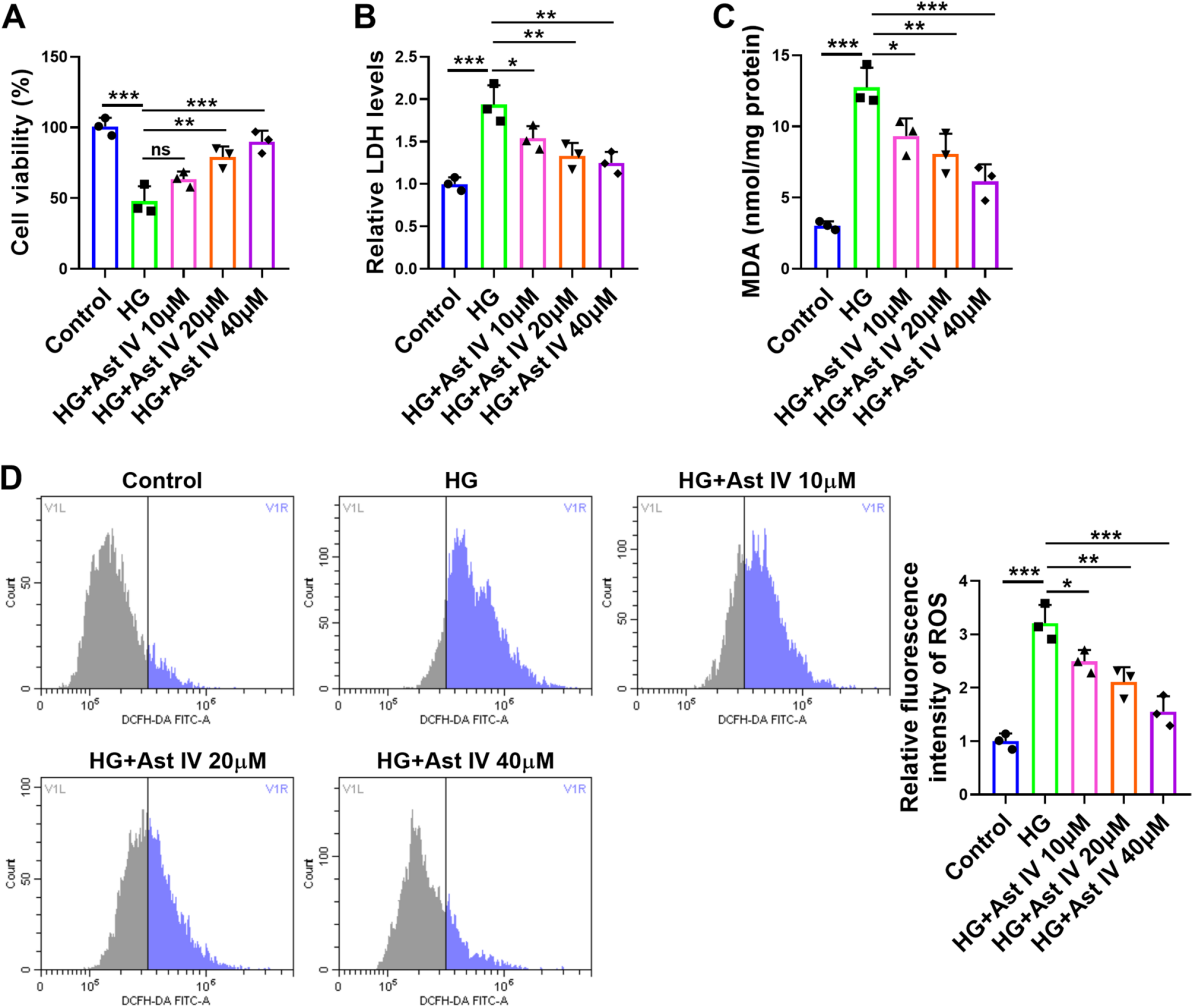
Ast IV enhances the proliferative capacity and attenuates oxidative stress in HTR-8/SVneo cells under HG environment. HTR-8/SVneo cells were divided into five groups: Control, HG, HG + Ast IV 10 µM, HG + Ast IV 20 µM, and HG + Ast IV 40 µM. **A**: CCK8 assay was used to assess the viability of the cells. **B**: The amount of LDH in HTR-8/SVneo cells was detected by colourimetric assay. **C**: Colourimetric assay was used to detect the level of MDA in HTR-8/SVneo cells. **D**: Fluorescence assay was used to detect content of ROS in HTR-8/SVneo cells. * *P* < 0.05, ** *P* < 0.01, *** *P* < 0.001, and ns indicated insignificant. Sample size description: The number of replicates for each experiment was 3 (*n* = 3)

In order to investigate the protective role of Ast IV in HTR-8/SVneo cells, we observed the impact of Ast IV on cell damage induced by HG in HTR-8/SVneo cells. HG treatment reduced the viability of HTR-8/SVneo cells, and after the application of Ast IV, the cell viability increased with the concentration of Ast IV (Fig. 1A). Furthermore, HG treatment resulted in a significant increase in the levels of LDH, MDA, and ROS in HTR-8/SVneo cells, whereas after the administration of Ast IV, the levels of these substances decreased as the concentration of Ast IV increased (Fig. 1B-D). It was revealed by the CCK8 experiment that toxicity was induced in cells when the concentration of Ast IV exceeded 40µM. Based on this finding, the concentration range of 10–40 µM was chosen for subsequent experiments (Supplementary Fig. 1). Altogether, the aforementioned findings indicate that Ast IV can attenuate the inhibitory effect of HG on the proliferation of HTR-8/SVneo cells and decrease the level of HG-induced oxidative stress.

### Ast IV ameliorates HG-induced ferroptosis in HTR-8/SVneo cells

**Fig. 2:**
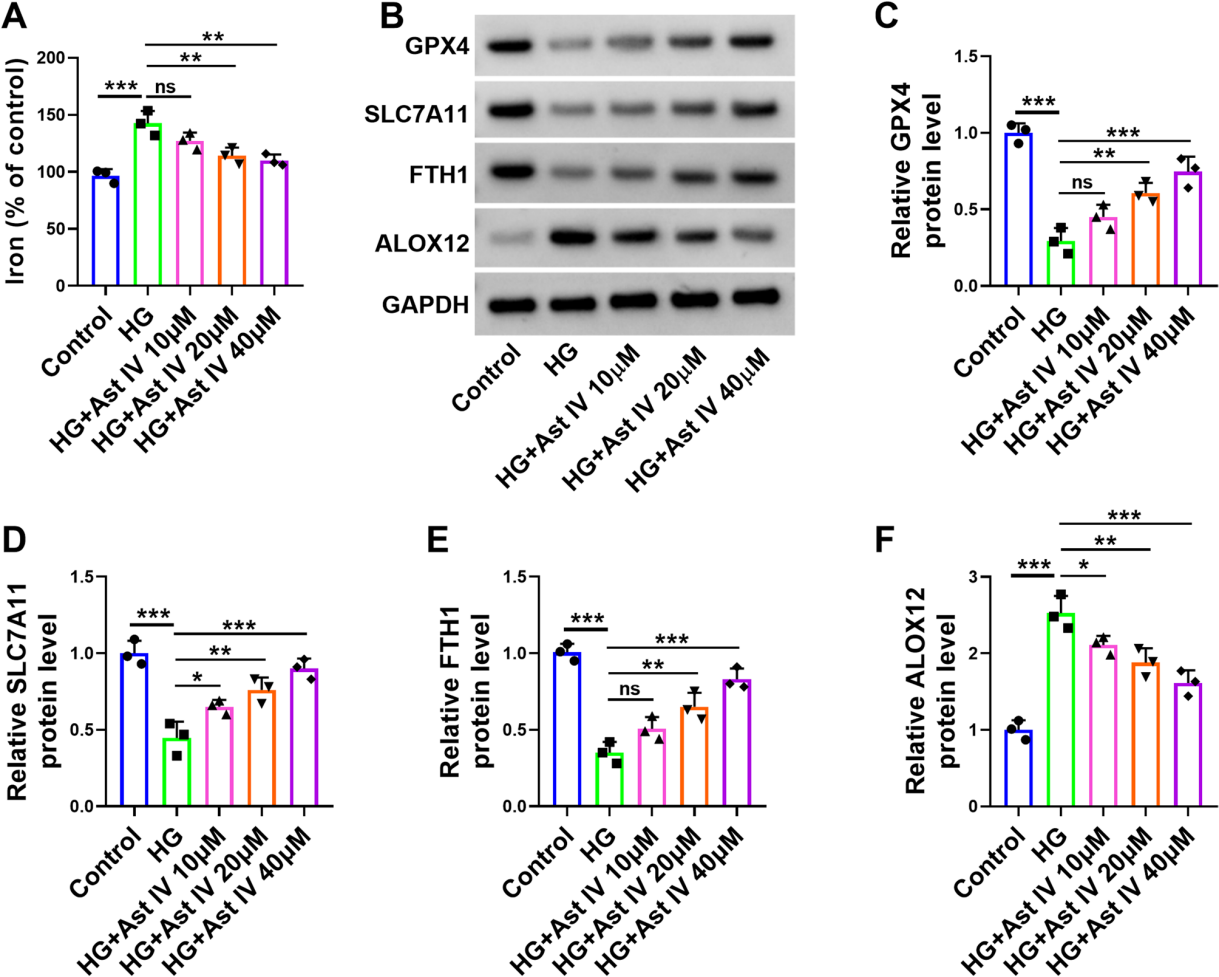
Ast IV is able to alleviate the HG-induced ferroptosis process in HTR-8/SVneo cells. HTR-8/SVneo cells were divided into five groups: Control, HG, HG + Ast IV 10 µM, HG + Ast IV 20 µM, and HG + Ast IV 40 µM. **A**: Colorimetric assay was used to detect the level of iron in HTR-8/SVneo cells. **B**-**F**: Western blot was used to demonstrate the expression levels of GXP4, SLC7A11, FTH1, and ALOX12 in HTR-8/SVneo cells. * *P* < 0.05, ** *P* < 0.01, *** *P* < 0.001, and ns indicated insignificant. Sample size description: The number of replicates for each experiment was 3 (*n* = 3)

To further investigate whether the protective effect of Ast IV on HTR-8/SVneo cells is related to ferroptosis, we evaluated the ferroptosis indicator of the cells. The findings indicated HG treatment led to a significant increase in iron content within HTR-8/SVneo cells, whereas upon the application of Ast IV, the iron content decreased as the concentration of Ast IV rose (Fig. 2A). Western blot results showed that while HG treatment significantly reduced the expression levels of GXP4, SLC7A11, and FTH1 in HTR-8/SVneo cells, the administration of Ast IV subsequently increased their expression levels in direct proportion to the amount added (Fig. 2B-E). Additionally, HG treatment increased the expression level of ALOX12, but after the administration of Ast IV, the expression level of ALOX12 decreased as the amount of Ast IV added increased (Fig. 2F). GPX4, SLC7A11, FTH1, and ALOX12 were the core genes involved in the ferroptosis pathway, in particular, the upregulation of GPX4, SLC7A11, and FTH1 effectively inhibited the progression of ferroptosis, whereas ALOX12 promoted ferroptosis [30]. In conclusion, Ast IV inhibits HG-induced ferroptosis in HTR-8/SVneo cells.

### Ast IV stabilizes the expression of FOXM1 by promoting its deubiquitination

**Fig. 3:**
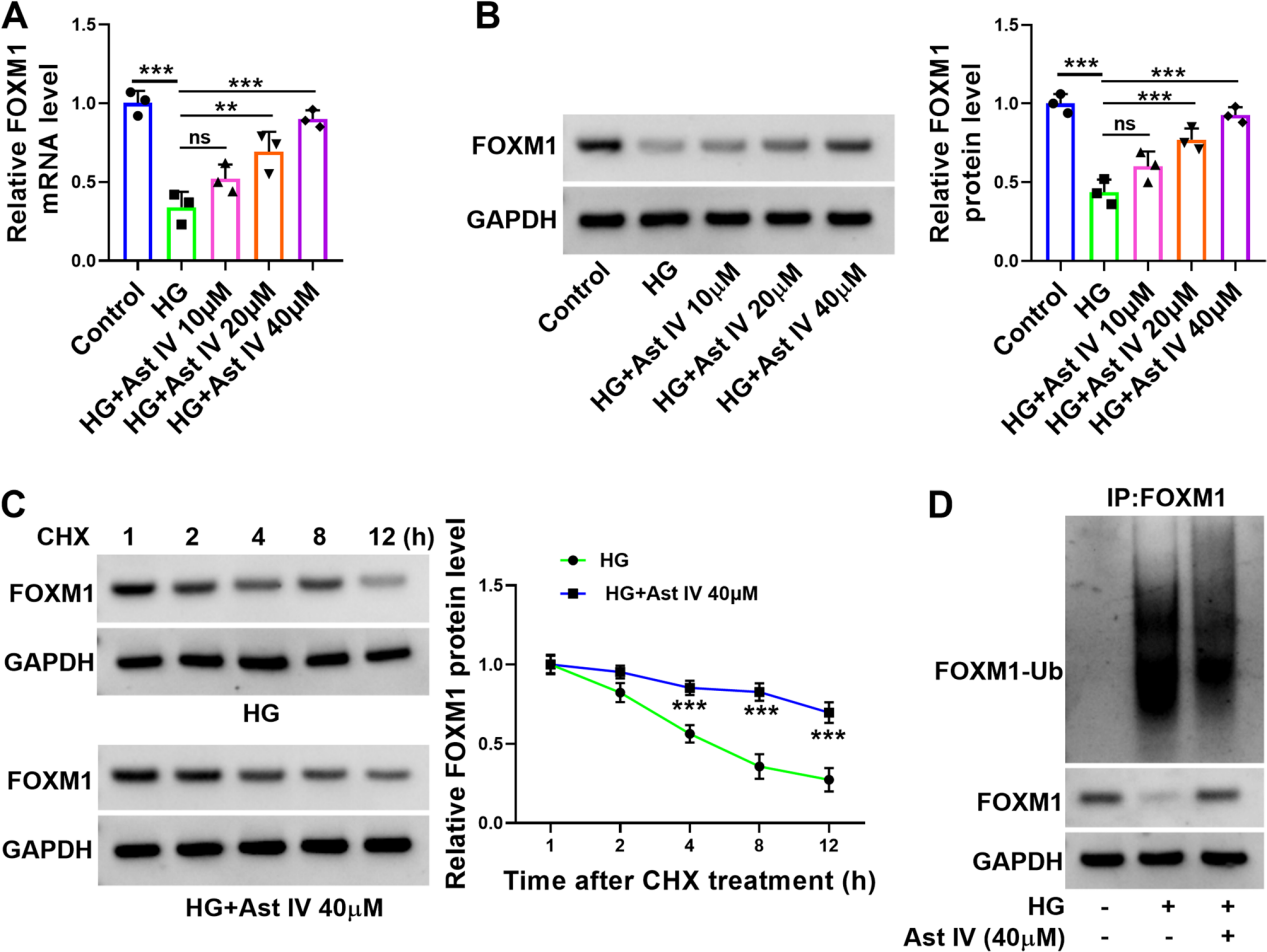
Ast IV reduces ubiquitination of FOXM1 and thus stabilizing its protein expression. HTR-8/SVneo cells were divided into five groups: Control, HG, HG + Ast IV 10 µM, HG + Ast IV 20 µM, and HG + Ast IV 40 µM. **A**: The mRNA expression level of FOXM1 in HTR-8/SVneo cells was detected by qRT-PCR. **B**: Western blot was used to assess the expression level of FOXM1 in HTR-8/SVneo cells. **C**: CHX assay was used to demonstrate the protein stability of FOXM1 in HTR-8/SVneo cells. **D**: The ubiquitination level of FOXM1 in HTR-8/SVneo cells was checked by immunoprecipitation assay. ** *P* < 0.01, *** *P* < 0.001, and ns indicated insignificant. Sample size description: The number of replicates for each experiment was 3 (*n* = 3)

Ubiquitination played a crucial role in regulating protein stability [23]. The qRT-PCR and western blot results demonstrated that HG treatment suppressed the expression of FOXM1 in HTR-8/SVneo cells, whereas the addition of Ast IV significantly elevated the expression level of FOXM1 in a dose-dependent manner (Fig. 3A-B). The addition of Ast IV significantly decreased the loss rate of FOXM1 protein (Fig. 3C). Then, we investigated the effects of Ast IV and HG on the polyubiquitination of FOXM1. Ast IV significantly reduced the polyubiquitination levels of FOXM1, while HG significantly accelerated the polyubiquitination levels of FOXM1 (Fig. 3D). When considered together, these results indicate that Ast IV inhibits HG-induced FOXM1 ubiquitination and stabilizes FOXM1 expression in HTR-8/SVneo cells.

### FOXM1 overexpression ameliorates HG-induced injury in HTR-8/SVneo cells

**Fig. 4:**
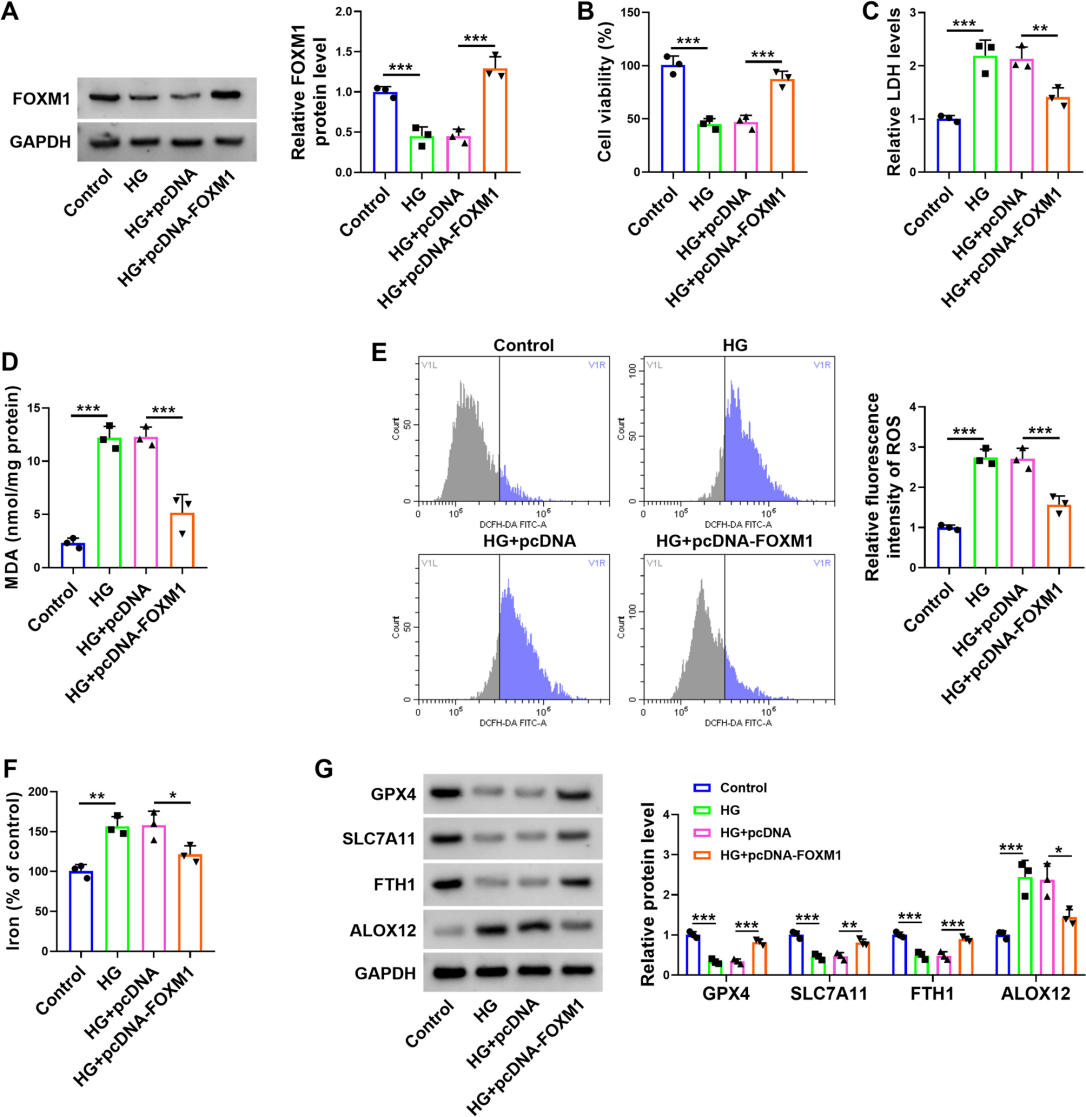
Overexpression of FOXM1 attenuates the damage caused by HG to HTR-8/SVneo cells. HTR-8/SVneo cells were divided into four groups: Control, HG, HG + pc-DNA, and HG + pc-DNA-FOXM1. **A**: Western blot was used to detect the expression level of FOXM1 in HTR-8/SVneo cells. **B**: The viability of the HTR-8/SVneo cells was assessed through CCK8 assay. **C**: Colourimetric assay was used to demonstrate the LDH content of the HTR-8/SVneo cells. **D**: The MDA level of the HTR-8/SVneo cells was detected by colourimetric assay. **E**: Fluorescence assay was used to detect ROS content in HTR-8/SVneo cells. **F**: The iron content in HTR-8/SVneo cells was detected through colourimetric assay. **G**: Western blot was used to demonstrate the expression levels of GXP4, SLC7A11, FTH1, and ALOX12 in HTR-8/SVneo cells. * *P* < 0.05, ** *P* < 0.01, and *** *P* < 0.001. Sample size description: The number of replicates for each experiment was 3 (*n* = 3)

Given the important role of FOXM1 in diabetes and its associated complications [31], we investigated the effects of FOXM1 in HG-induced cell injury. The western blot results showed that the expression level of FOXM1 was increased after HTR-8/SVneo cells were transfected with pcDNA-FOXM1 (Fig. 4A). The cell viability of HTR-8/SVneo cells was enhanced after pcDNA-FOXM1 transfection (Fig. 4B). Additionally, after HTR-8/SVneo cells were transfected with pcDNA-FOXM1, the LDH content, MDA content, ROS content, and iron content were reduced (Fig. 4C-F). The western blot results demonstrated that compared with the HG and HG + pcDNA groups, the protein expression levels of GXP4, SLC7A11, and FTH1 were increased in the HG + pcDNA-FOXM1 group, whereas the protein expression levels of ALOX12 were decreased (Fig. 4G). Collectively, our findings show that FOXM1 enhances the activity of HTR-8/SVneo cells and inhibits oxidative stress and ferroptosis under a HG environment.

### Ast IV ameliorates HG-induced injury in HTR-8/SVneo cells by modulating FOXM1

**Fig. 5:**
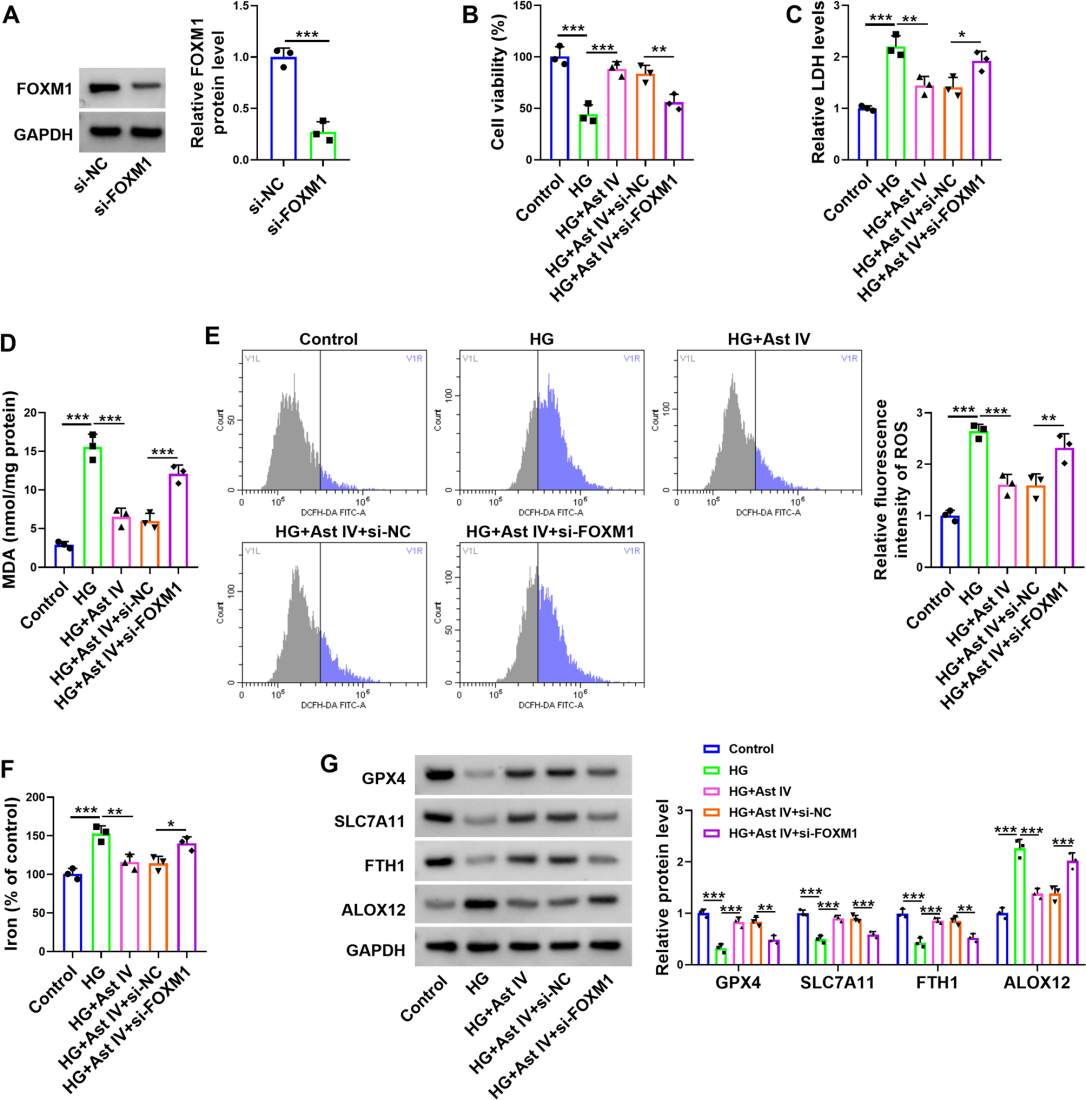
Knockdown of FOXM1 counteracts the effects of Ast IV. HTR-8/SVneo cells were divided into five groups: Control, HG, HG + Ast IV, HG + Ast IV + si-NC, and HG + Ast IV + si-FOXM1. **A**: Western blot was used detect the expression level of FOXM1 in HTR-8/SVneo cells. **B**: CCK8 assay was used to test the viability of the HTR-8/SVneo cells. **C**: The content of LDH in HTR-8/SVneo cells was checked through colourimetric assay. **D**: Colourimetric assay was used to detect MDA content in HTR-8/SVneo cells. **E**: The ROS content in HTR-8/SVneo cells was tested by fluorescence assay. **F**: Colourimetric assay was used to detect iron content in HTR-8/SVneo cells. **G**: Western blot was used to detect the expression levels of GXP4, SLC7A11, FTH1, and ALOX12 in HTR-8/SVneo cells. * *P* < 0.05, ** *P* < 0.01, and *** *P* < 0.001. Sample size description: The number of replicates for each experiment was 3 (*n* = 3)

To investigate whether the effects mediated by Ast IV in HTR-8/SVneo cells depended on FOXM1, we attempted to establish a si-FOXM1 cell line. The western blot results showed that compared with the si-NC group, the protein expression levels of FOXM1 were reduced in the si-FOXM1 group (Fig. 5A). Ast IV alleviated the inhibitory effect of HG treatment on the viability of HTR-8/SVneo cells, while knockdown of FOXM1 partially reversed this mitigating effect of Ast IV (Fig. 5B). HG treatment elevated LDH, MDA, ROS, and iron levels in HTR-8/SVneo cells, but Ast IV reversed this increase, and knocking down FOXM1 partially attenuated Ast IV’s reversing effect (Fig. 5C-F). Western blot results showed that HG treatment in HTR-8/SVneo cells suppressed GXP4, SLC7A11, and FTH1 expression while enhancing ALOX12 expression, with Ast IV partially reversing these effects but knocking down FOXM partially counteracting Ast IV’s reversal (Fig. 5G). These results indicate that the damage caused by HG to HTR-8/SVneo cells is suppressed by Ast IV through increasing the expression level of FOXM1.

## Discussion

Trophoblast cells occupy a central position in placental development and functional maintenance, and their dysfunction may lead to placental abnormalities, subsequently causing a series of pregnancy complications [32]. GDM, as a common metabolic disorder, poses a significant threat to maternal and fetal health [33, 34]. Although the pathological mechanisms of GDM have been extensively studied, the dysfunction of trophoblast cells under HG conditions and their association with pregnancy complications have not received adequate attention [35, 36]. Therefore, it is of great significance to delve into the correlation between trophoblast cell dysfunction under high-glucose conditions and pregnancy complications related to GDM. Research suggests that a HG environment can induce dysfunction in trophoblast cells, which subsequently triggers placental vascular endothelial injury and inflammatory responses, activating the renin-angiotensin-aldosterone system, resulting in vasoconstriction, elevated blood pressure, and ultimately causing gestational hypertension disorders [37, 38]. Additionally, a HG environment might also affect trophoblast cell function and cause placental developmental abnormalities by inhibiting the expression and nuclear translocation of β-catenin in the Wnt signaling pathway [39, 40]. In this study, the trophoblast cell line HTR-8/SV was utilized as a model, and it was found that high-glucose exposure significantly inhibited the proliferation of HTR-8/SV cells and induced oxidative stress and ferroptosis.

In traditional Chinese medicine, Ast IV is frequently employed as a therapeutic component for addressing viral and bacterial infections, inflammatory conditions, as well as malignancies [41]. For instance, Ast IV inhibited lipolysis by regulating the Akt/PDE3B signaling pathway, effectively reducing the accumulation of cAMP, which aided in reducing hepatic lipid deposition and controlling the overproduction of hepatic glucose [42]. In a diabetes rat model, Ast IV efficiently blocked cellular damage caused by elevated glucose concentrations by enhancing the production of miR-128 [43]. In the study, we noted that Ast IV had the ability to reduce the suppressive impact of HG on the growth of HTR-8/SVneo cells. It also alleviated the oxidative stress induced by HG and effectively inhibited the ferroptosis process in HTR-8/SVneo cells. This indicated that Ast IV is able to attenuate the damage caused by HG to HTR-8/SVneo cells. Although our study has provided conclusive evidence for the cytoprotective effect of Ast IV against HG-induced damage in HTR-8/SVneo cells, its potential applications in animal models and clinical settings still warrant in-depth investigation. Future research should focus on elucidating the specific mechanisms of Ast IV in vivo, evaluating its safety and efficacy in animal models, and conducting clinical trials to assess its therapeutic potential in HG-related diseases.

Ubiquitination, as a key post-translational modification process, plays a central role in eukaryotic cells, primarily through its fine regulation of functional properties, intracellular positional distribution, interactions with other proteins, and stability of the modification target [23]. In this study, we found that Ast IV reduced the ubiquitination level of FOXM1 in HTR-8/SVneo cells, thereby stabilizing its expression. Further research indicated that FOXM1 overexpression not only alleviated the inhibition of HTR-8/SVneo cell growth induced by HG but also effectively counteracted HG-induced oxidative stress and significantly inhibited the process of cellular ferroptosis. Furthermore, reducing FOXM1 expression under HG conditions weakened the protective effect of Ast IV on HTR-8/SVneo cells. It indicated that Ast IV exert a notable ameliorative effect on HG-induced cellular injury by inhibiting the ubiquitination process of FOXM1 in HTR-8/SVneo cells.

Ubiquitination, a sophisticated and precisely regulated protein modification mechanism mediated by a sequential cascade of ubiquitin-activating enzymes (E1), ubiquitin-conjugating enzymes (E2), and ubiquitin ligases (E3), relies on deubiquitinating enzymes (DUBs) to maintain its dynamic equilibrium throughout the process [44, 45]. During the process of Ast IV inhibiting FOXM1 ubiquitination, specific E3 ligases may mediate the transfer of ubiquitin molecules to FOXM1, leading to an increase in its ubiquitination level, while Ast IV may reduce FOXM1 ubiquitination by inhibiting the activity of these E3 ligases [46, 47]. On the other hand, DUBs may restore FOXM1 stability by hydrolyzing ubiquitin chains on FOXM1, and Ast IV may enhance the hydrolytic effect of certain deubiquitinating enzymes, such as USP7, on FOXM1 ubiquitin chains, thereby decreasing FOXM1 ubiquitination levels and stabilizing its expression [48, 49]. However, the precise mechanisms by which Ast IV interacts with E3 ligases and deubiquitinating enzymes, as well as the specific enzymes involved in this process, remain unclear and warrant further investigation.

In the field of oncology research, the transcription factor FOXM1 is extensively documented as a regulator of numerous downstream genes, which are involved in various aspects such as cell cycle regulation ( CCNB1, CCNB2), angiogenesis (VEGF), and antioxidant defense (SOD) [50, 51].Taking breast cancer as an example, FOXM1’s promotion of intratumoral blood vessel generation through upregulating VEGF expression unveiled its pivotal role in tumor progression [52]. Given that FOXM1 could regulate angiogenesis and antioxidant defense, it was speculated that it might influence placental vascular function in GDM through similar mechanisms, but there was no direct evidence to indicate whether it regulated the expression of genes such as CCNB1, VEGF, or SOD in GDM [52, 53]. Nevertheless, based on research on FOXM1 in other diseases, it was reasonable to hypothesize that in GDM, FOXM1 might participate in the disease process by regulating the expression of downstream genes such as VEGF and SOD [54, 55]. Future studies should have focused on verifying this hypothesis, namely, whether FOXM1 indeed influenced disease progression in GDM through the regulation of its downstream genes. Additionally, exploring the potential of FOXM1 as a therapeutic target for GDM would also provide important clues for the development of novel treatment strategies.

## Conclusion

In summary, Ast IV is capable of alleviating trophoblast cell damage caused by HG through inhibiting the ubiquitination process of FOXM1. This study has made an important breakthrough in the field of GDM research, revealing for the first time the research gap of FOXM1 in this field and, at the same time, deeply exploring and elucidating the novel mechanism of Ast IV regulating ubiquitination. This finding provides a new direction for the study of the pathogenesis of GDM and lays a solid theoretical foundation for the development of potential therapeutic strategies for GDM.

## Electronic supplementary material

Below is the link to the electronic supplementary material.

Supplementary Material 1

Supplementary Material 2

Supplementary Material 3

## Data Availability

No datasets were generated or analysed during the current study.
