# Supplementary material for: Astragaloside IV regulates FOXM1 deubiquitination to ameliorate trophoblast damage caused by high glucose
Source: Hereditas. 2025 Jun 13;162:104. doi: 10.1186/s41065-025-00465-w (PMC12166594; doi:10.1186/s41065-025-00465-w)
Supplement: Supplementary file 1 — Supplementary Material 1 [file 41065_2025_465_MOESM1_ESM.docx]

Supplementary table 1 RT-qPCR reaction program

| 温度 | 时间 |
| --- | --- |
|  |  |
| 94℃ | 30min |
| 94℃ | 5s |
| 60℃ | 30s (35个循环） |
| 94℃ | 15s |
| 60℃ | 1min |
| 94℃ | 15s |
