# Supplementary figures and images for: Astragaloside IV regulates FOXM1 deubiquitination to ameliorate trophoblast damage caused by high glucose
Source: Hereditas. 2025 Jun 13;162:104. doi: 10.1186/s41065-025-00465-w (PMC12166594; doi:10.1186/s41065-025-00465-w)

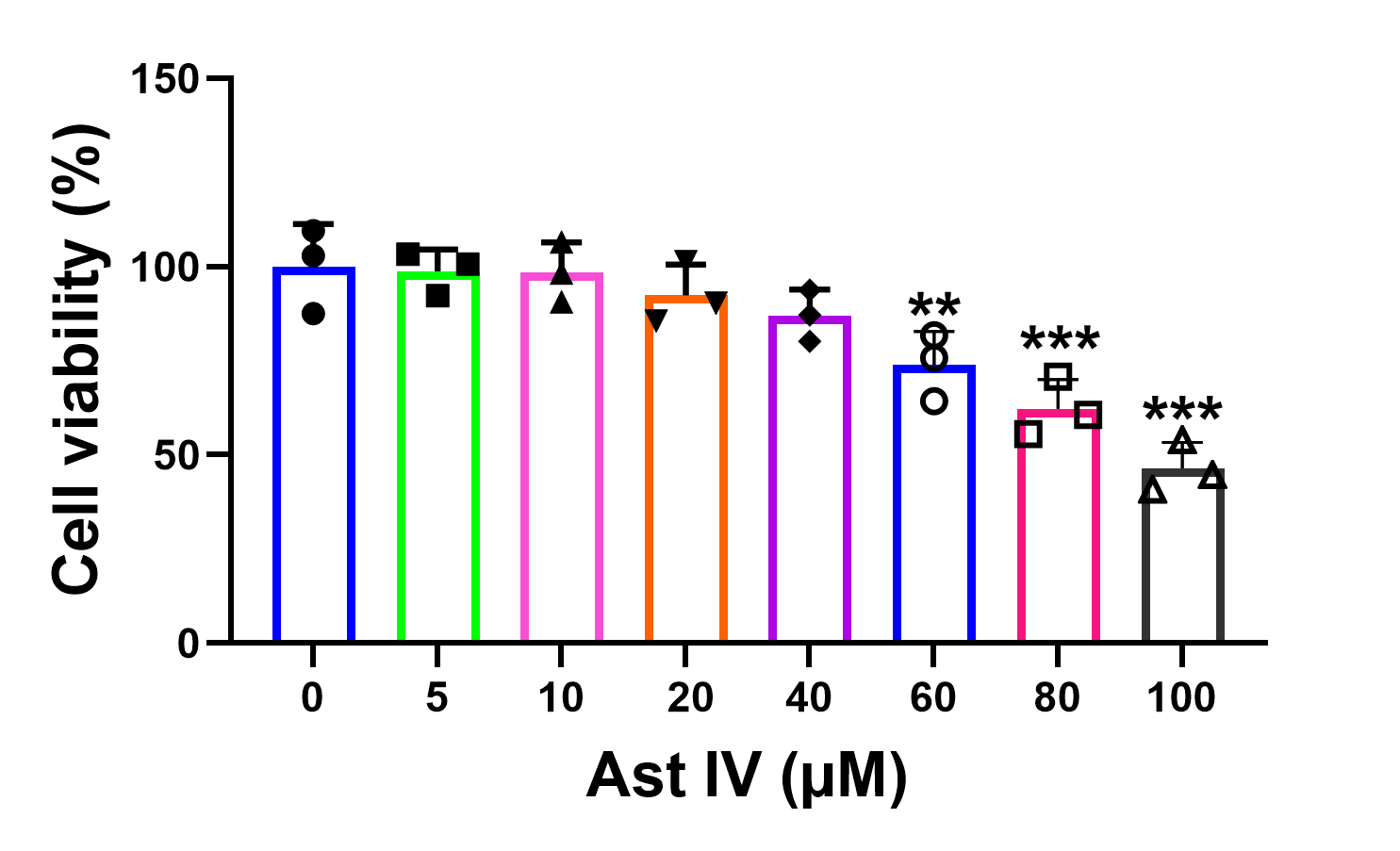

Supplement: Supplementary file 2 — Supplementary Material 2 [file 41065_2025_465_MOESM2_ESM.tif]
