# Supplementary material for: Astragaloside IV regulates FOXM1 deubiquitination to ameliorate trophoblast damage caused by high glucose
Source: Hereditas. 2025 Jun 13;162:104. doi: 10.1186/s41065-025-00465-w (PMC12166594; doi:10.1186/s41065-025-00465-w)

Fig2B

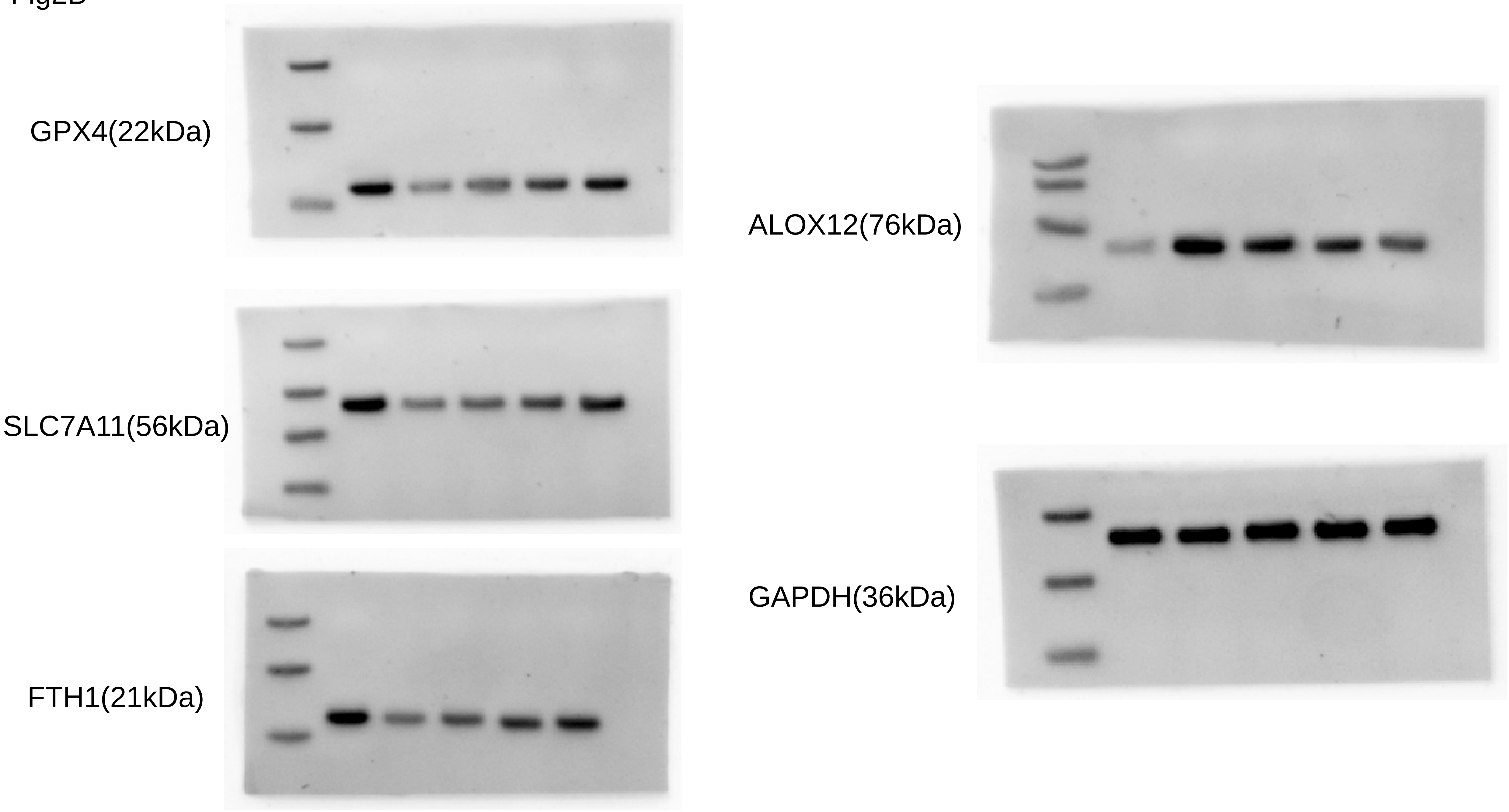
GPX4(22kDa)
SLC7A11(56kDa)
FTH1(21kDa)
ALOX12(76kDa)
GAPDH(36kDa)

Fig3B

FOXM1(89kDa)

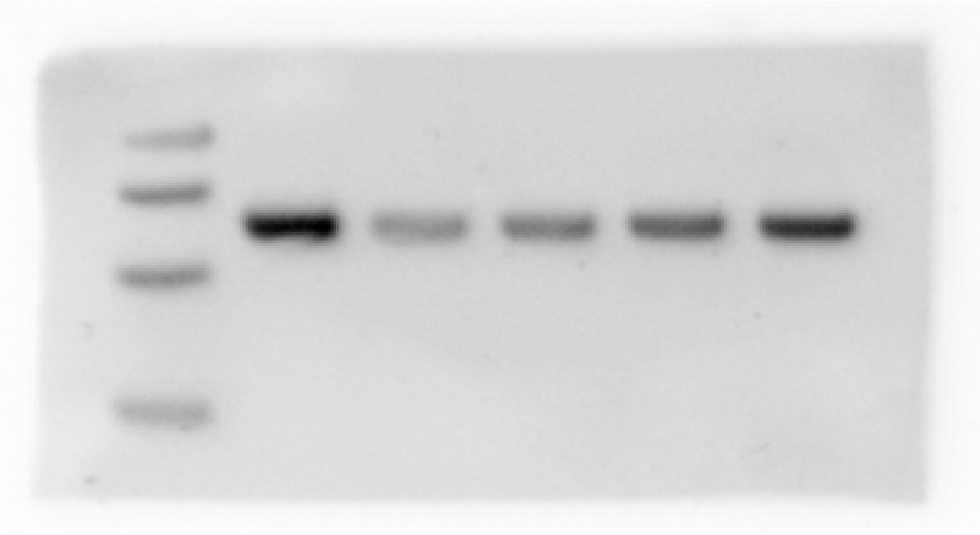

GAPDH(36kDa)

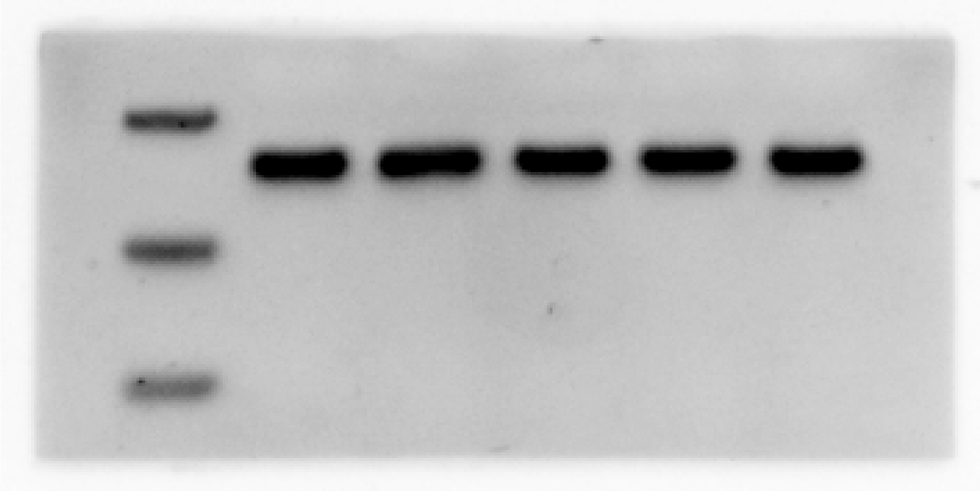

Fig3C

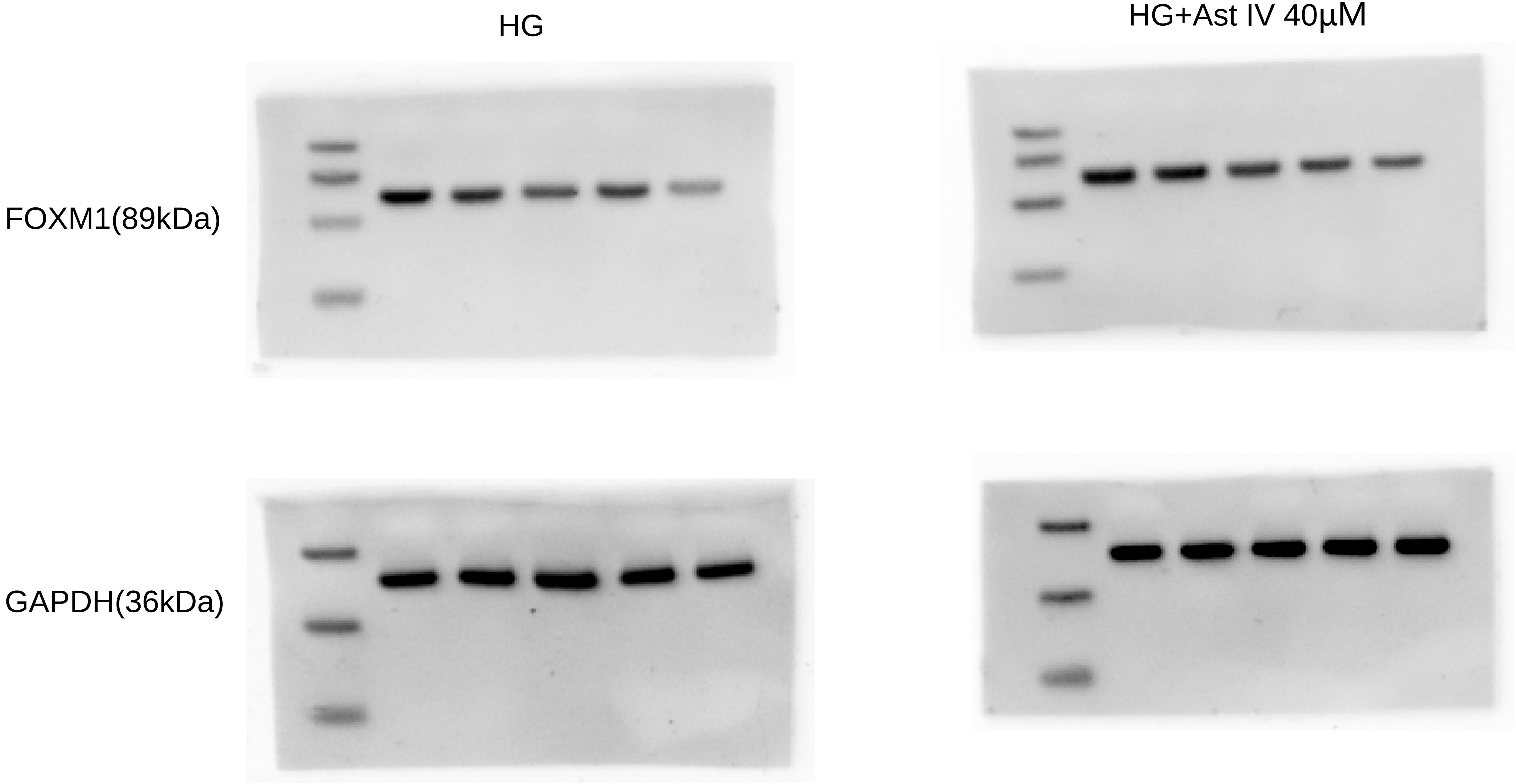

Fig3D

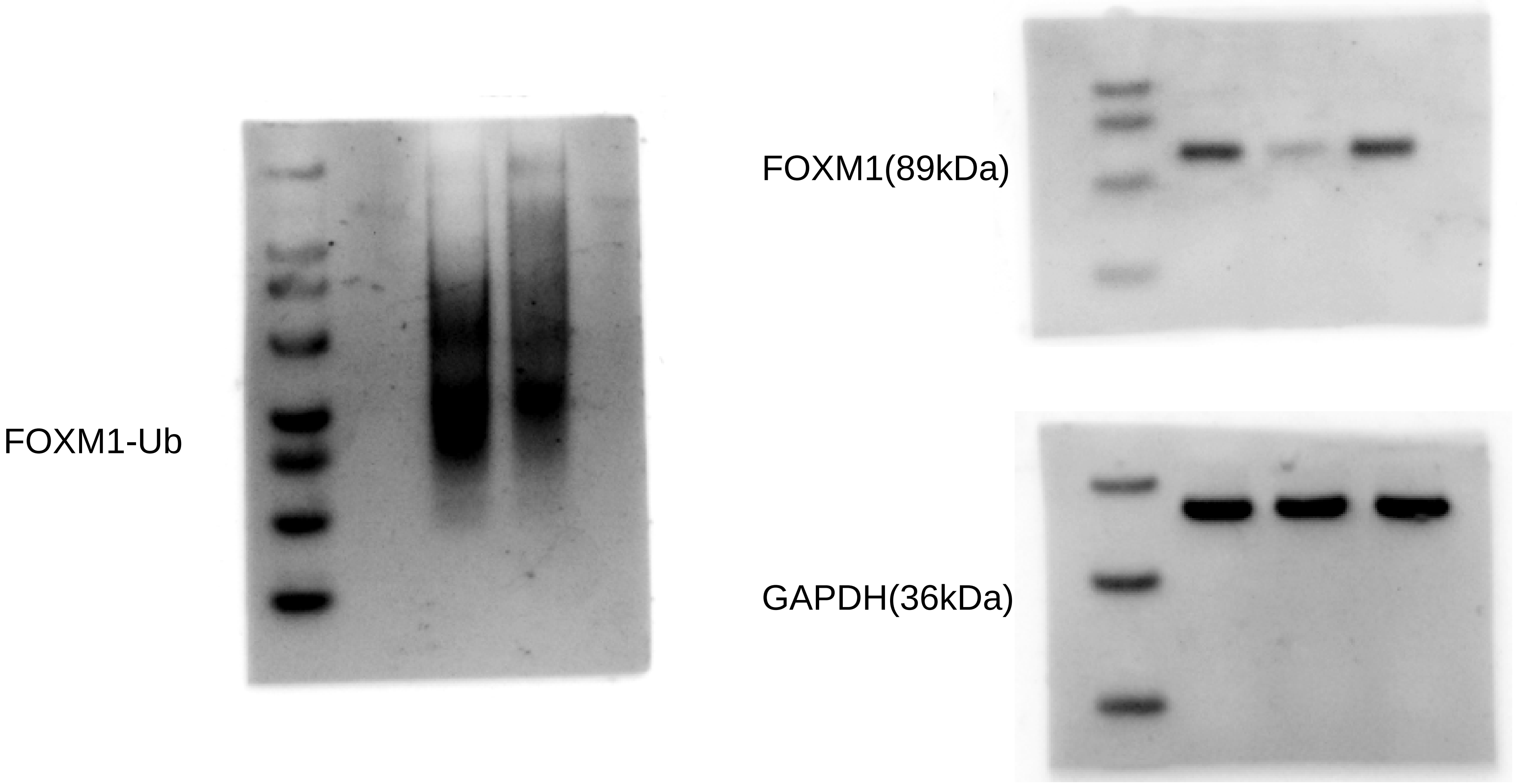

Fig4A

FOXM1(89kDa)

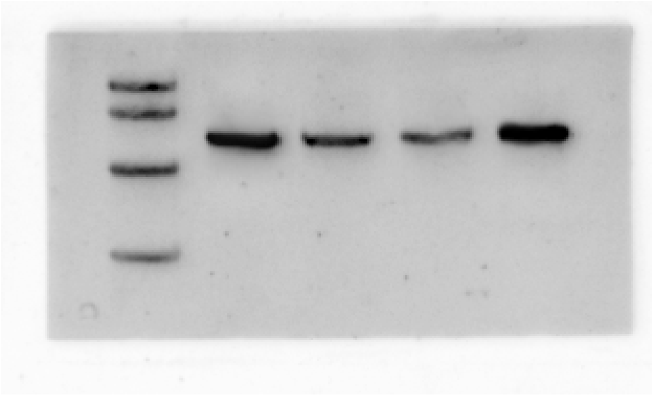

GAPDH(36kDa)

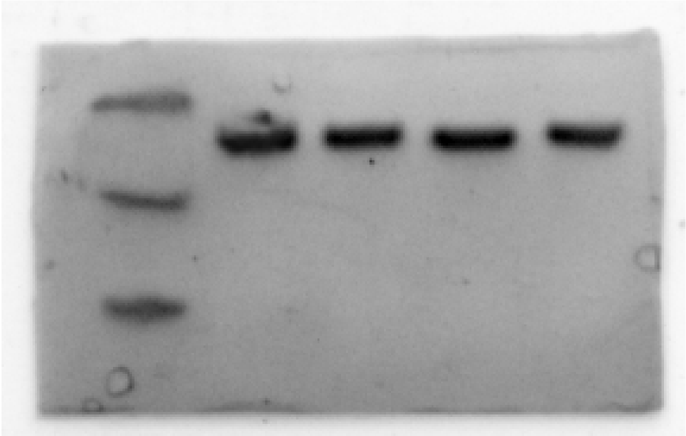

Fig4G

GPX4(22kDa)

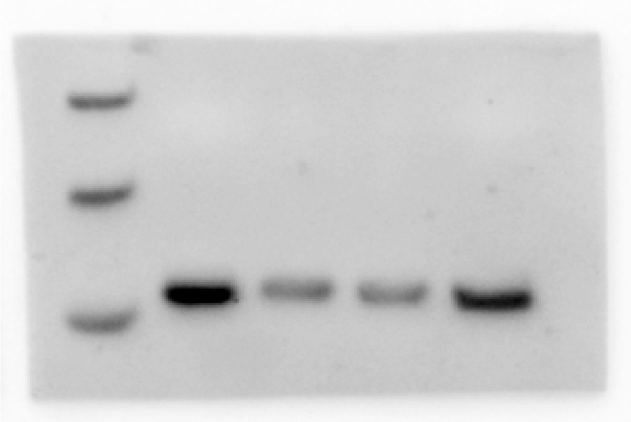

SLC7A11(56kDa)

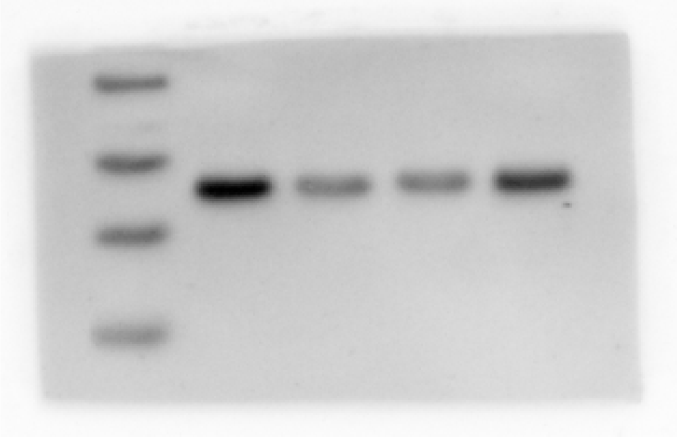

FTH1(21kDa)

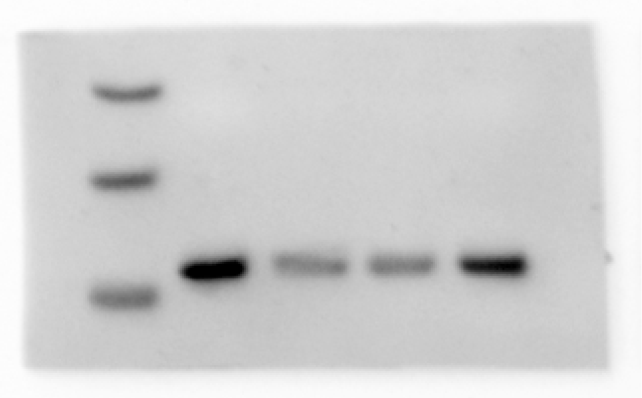

ALOX12(76kDa)

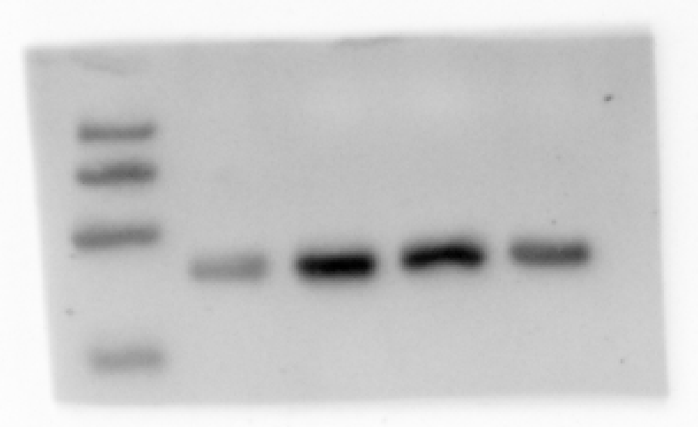

GAPDH(36kDa)

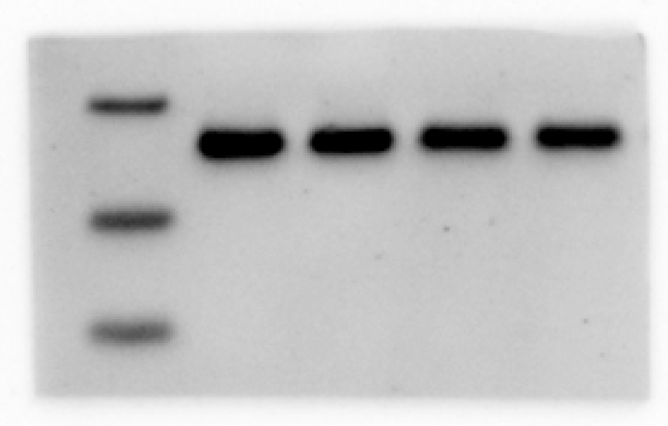

Fig5A

FOXM1(89kDa)

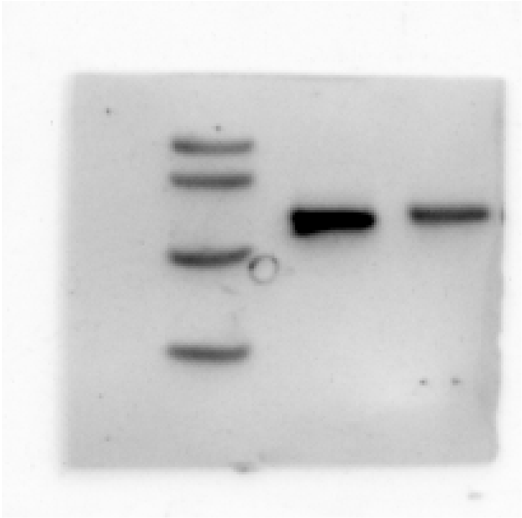

GAPDH(36kDa)

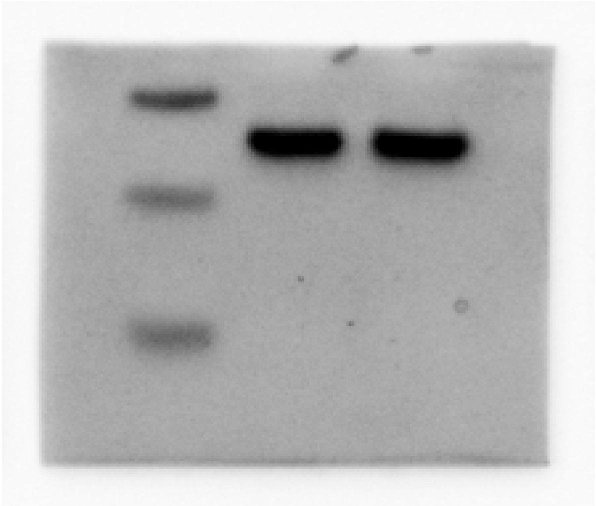

Fig5G

GPX4(22kDa)

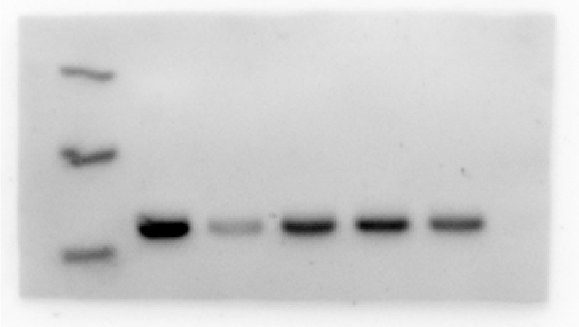

SLC7A11(56kDa)

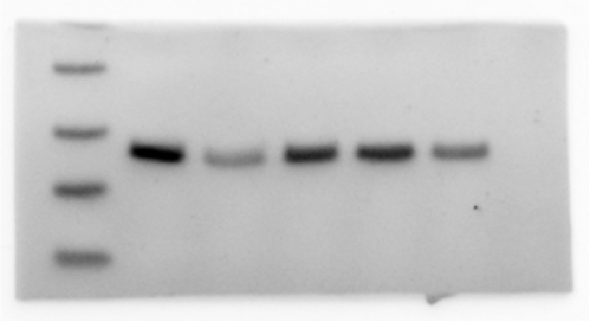

FTH1(21kDa)

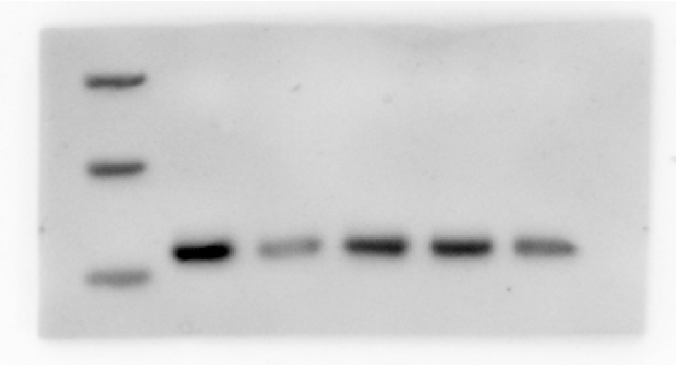

ALOX12(76kDa)

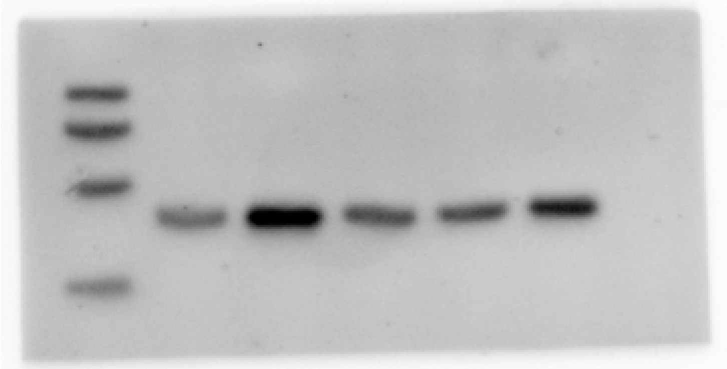

GAPDH(36kDa)

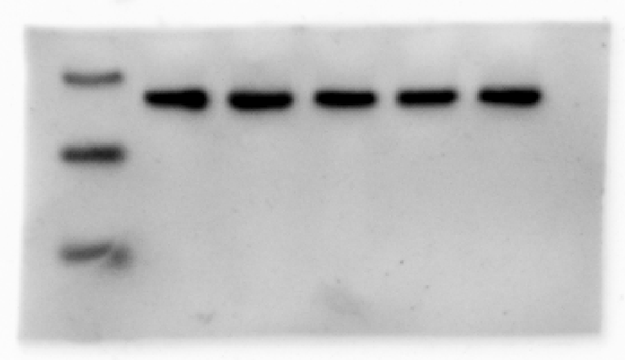

marker

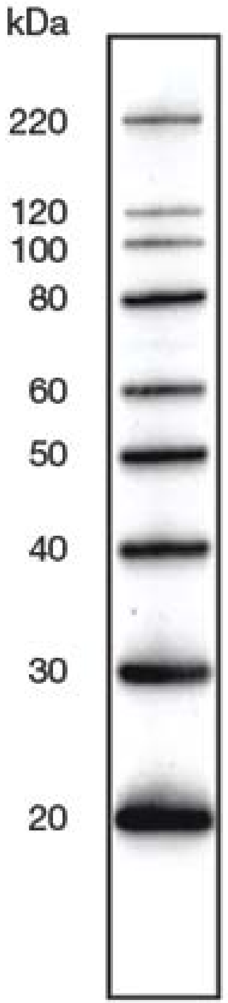

Supplement: Supplementary file 3 — Supplementary Material 3 [file 41065_2025_465_MOESM3_ESM.pdf]
